# Durlobactam, a New Diazabicyclooctane β-Lactamase Inhibitor for the Treatment of *Acinetobacter* Infections in Combination With Sulbactam

**DOI:** 10.3389/fmicb.2021.709974

**Published:** 2021-07-19

**Authors:** Adam B. Shapiro, Samir H. Moussa, Sarah M. McLeod, Thomas Durand-Réville, Alita A. Miller

**Affiliations:** Entasis Therapeutics, Waltham, MA, United States

**Keywords:** *Acinetobacter*, durlobactam, sulbactam, DBO, β-lactamase inhibitor

## Abstract

Durlobactam is a new member of the diazabicyclooctane class of β-lactamase inhibitors with broad spectrum activity against Ambler class A, C, and D serine β-lactamases. Sulbactam is a first generation β-lactamase inhibitor with activity limited to a subset of class A enzymes that also has direct-acting antibacterial activity against *Acinetobacter* spp. The latter feature is due to sulbactam’s ability to inhibit certain penicillin-binding proteins, essential enzymes involved in bacterial cell wall synthesis in this pathogen. Because sulbactam is also susceptible to cleavage by numerous β-lactamases, its clinical utility for the treatment of contemporary *Acinetobacter* infections is quite limited. However, when combined with durlobactam, the activity of sulbactam is effectively restored against these notoriously multidrug-resistant strains. This sulbactam-durlobactam combination is currently in late-stage development for the treatment of *Acinectobacter* infections, including those caused by carbapenem-resistant isolates, for which there is a high unmet medical need. The following mini-review summarizes the molecular drivers of efficacy of this combination against this troublesome pathogen, with an emphasis on the biochemical features of each partner.

## Introduction

Infections caused by multi-drug resistant (MDR) *Acinetobacter* species are among the most urgent threats to human health [Global Action Plan on Antimicrobial Resistance, 2014; Centers for Disease Control and Prevention (CDC), 2019]. These pathogens cause hospital-acquired or ventilator-associated pneumonia (VAP), bacteremia, complicated urinary tract infections and a variety of skin and tissue infections, in both healthy and immuno-compromised individuals (Lee et al., 2017). Most *Acinetobacter* infections are chronic, with mortality rates of 40–60% (Wong et al., 2017). The incidence of these infections varies widely across the globe, ranging from 1% of surgical site infections and 12% of VAP in the United States (Weiner et al., 2016) to 35% of all hospital-acquired drug-resistant infections in China (Zhang et al., 2019). Clinical resistance in this organism to nearly all antibiotic classes, including cephalosporins, fluoroquinolones, aminoglycosides, and tetracyclines, is widespread and continues to increase (Magiorakos et al., 2012; Wong et al., 2017). In the past few decades, resistance to carbapenems in *Acinetobacter* and even the last resort agent colistin, has also increased at alarming rates worldwide (Lee et al., 2017).

Despite the significant unmet medical need, there are currently no reliably effective antibiotics for the treatment of carbapenem-resistant *Acinetobacter* infections. Although cefiderocol (Fetroja^®^), which was recently approved for the treatment of drug-resistant Gram-negative pathogens, has potent *in vitro* activity against MDR *Acinetobacter* (Isler et al., 2019), its *in vivo* efficacy in preclinical infection models of infection against cefiderocol-susceptible *Acinetobacter baumannii* is quite variable (Monogue et al., 2017). In addition, treatment with this agent resulted in higher mortality rates as compared to best available therapy in patients with *A. baumannii* bloodstream infections or nosocomial pneumonia in the recent CREDIBLE-CR Phase 3 trial (Bassetti et al., 2021). These findings have recently been proposed to be related to liabilities associated with siderophore-mediated uptake leading to heteroresistance (Choby et al., 2021).

The only agent currently in late-stage clinical development for this indication is a combination of sulbactam, a first generation β-lactamase inhibitor (BLI) with intrinsic antibacterial activity against *Acinetobacter* spp., plus durlobactam, a next generation diazabicyclooctane (DBO) β-lactamase inhibitor with broad-spectrum activity against Class A, C, and D β-lactamases (Durand-Reville et al., 2017). The key features of this unusual, dual BLI combination therapy are described below.

## Sulbactam: A β-Lactamase Inhibitor With Intrinsic Antibacterial Activity Against *Acinetobacter*

Sulbactam is a semi-synthetic penicillanic acid that was among the first β-lactamase inhibitors developed, in combination with ampicillin, for the treatment of infections caused by β-lactamase-producing bacterial pathogens (Adnan et al., 2013). Its inhibitory activity is limited to a subset of class A serine β-lactamases (Shapiro, 2017). A unique feature of sulbactam is its intrinsic antibacterial activity against *Acinetobacter* and a limited number of other bacterial species (Noguchi and Gill, 1988), which results from its inhibition of key enzymes required for bacterial peptidoglycan synthesis. PBP1a, PBP1b, and PBP3, but not PBP2, are targets of sulbactam in *Acinetobacter* species. This was shown by its selectivity of inhibition of BOCILLIN FL penicillin labeling in membranes prepared from *A. baumannii*, and by sulbactam-induced cell filamentation, a hallmark of Gram-negative PBP1/PBP3 inhibition (Penwell et al., 2015).

**FIGURE 1:**
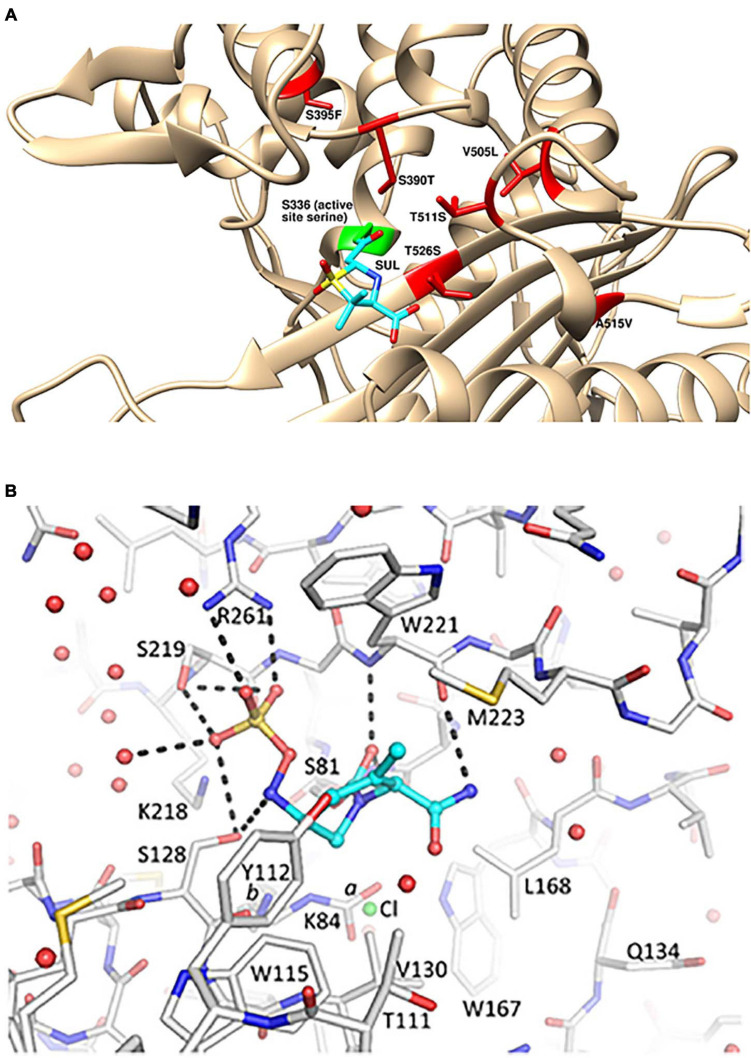
Structural details for the mechanism of action of sulbactam-durlobactam. **(A)** Computational model of sulbactam bound to the active site of *Acinetobacter baumannii* PBP3 (PDB: 3UE3). Covalent docking was performed in ICM-Pro version 3.8 and structure visualized in UCSF Chimera version 1.15 (Pettersen et al., 2004), with sulbactam bound to the PBP3 active site serine, shown in green. Sulbactam-resistant mutations are indicated in red and sulbactam is shown in cyan. **(B)** Durlobactam bound to the active site of OXA24/40 (PDB: 6MPQ). The inhibitor is shown in blue carbon atom stick representation, whereas the protein is depicted in gray carbon stick representation. The K84 side chain was observed in two conformations: one carbamylated and one non-carbamylated [0.6 and 0.4 occupancy conformations labeled a and b, respectively]. A chloride ion with 0.4 occupancy was also refined in the active site (green sphere). Hydrogen bonds between durlobactam and OXA24/40 are shown with dashed lines. Water molecules are depicted as red spheres. Reproduced from Barnes et al. (2019) with permission from the authors.

Spontaneous resistance mutants to sulbactam selected at a very low frequency *in vitro* mapped to mutations in PBP3 near the active site (Penwell et al., 2015; McLeod et al., 2018). These included S390T, S395F, V505L, and T511A/S mutations (Figure 1A). The S390T, S395F, and T511S mutations reduced the potency of inhibition by sulbactam by over 90%, as measured by *k_*inact*_/K_*i*_* (McLeod et al., 2018). *A. baumannii* strains with the S390T and S395F mutations in PBP3 had markedly reduced growth rates *in vitro* suggesting that *A. baumannii* bearing those sulbactam resistance mutations may exhibit reduced virulence *in vivo* (Penwell et al., 2015).

Several decades ago, sulbactam demonstrated both *in vitro* activity and clinical effectiveness against *A. baumannii* isolates (Doi et al., 2015). However, there has been a steady decline in the *in vitro* susceptibility of *A. baumannii* to sulbactam since that time, due to its susceptibility to degradation by a variety of acquired or upregulated β-lactamases, including Ambler class A TEM-1, class C ADC-30 and several class D OXAs (Krizova et al., 2013; Kuo et al., 2015). Indeed, the need for a β-lactamase-resistant β-lactamase inhibitor in combination with sulbactam for the treatment of *A. baumannii* infections is clearly shown by the fact that β-lactamases of all 4 Ambler classes are able to degrade sulbactam, whereas only a subset of class A β-lactamases are potently inhibited by it (Shapiro, 2017).

## Durlobactam: A Potent, Broad-Spectrum DBO Inhibitor of Class A, C, and D β-Lactamases

Durlobactam is a next generation DBO β-lactamase inhibitor with an extended spectrum of activity compared to other β-lactamase inhibitors currently on the market. It was discovered using structure-based drug design, computational chemistry and medicinal chemistry with a design hypothesis based on a combination of increased chemical reactivity, improved enzymatic binding, optimized Gram-negative permeation and physico-chemical properties suitable for intravenous dosing (Durand-Reville et al., 2017).

As shown in Table 1, durlobactam is a potent inhibitor of class A, C, and D serine β-lactamases. The key differentiating feature as compared to other DBO BLIs is its activity against class D carbapenemases of the OXA family, which are prevalent in *A. baumannii* (Durand-Reville et al., 2017). Durlobactam does not inhibit class B metallo-β-lactamases, but recent surveillance studies suggest these are currently rare in global clinical isolates of *Acinetobacter* spp. (Butler et al., 2019; Kostyanev et al., 2021).

**TABLE 1:** *k_*inact*_/K_i_* and *k*_*off*_ of durlobactam and *k_*inact*_/K_*i*_* for avibactam with β-lactamases^a^.

**Class**	**β -Lactamase**	**Durlobactam**	**Durlobactam**	**Avibactam**
		***k_*inact*_/K_*i*_* (M**^–^**^1^ s**^–^**^1^)**	***k*_*off*_ (s**^–^**^1^)**	***k_*inact*_/K_*i*_* (M**^–^**^1^ s**^–^**^1^)**
A	CTX-M-15	7 (±2) × 10^6^	2.2 (±0.5) × 10^–4^	8 × 10^5^
A	SHV-5	6.4 (±0.5) × 10^6^	5.5 (±0.3) × 10^–4^	1 × 10^5^
A	TEM-1	1.4 (±0.6) × 10^7^	1.4 (±0.2) × 10^–3^	4 × 10^5^
A	KPC-2	9.3 (±0.6) × 10^5^	1.0 (±0.1) × 10^–3^	6 × 10^3^
A	KPC-3	8 (±1) × 10^5^	2.7 (±0.7) × 10^–4^	7.1 (± 0.7) × 10^3^
C	*Pseudomonas aeruginosa* AmpC	9 (±5) × 10^5^	4 (±1) × 10^–3^	3 × 10^3^
C	*Enterobacter cloacae* P99	2.3 (±0.4) × 10^6^	3.4 (±0.1) × 10^–4^	8 × 10^3^
C	*Acinetobacter baumannii* ADC-7	1.0 (±0.1) × 10^6^	8 (±1) × 10^–4^	NT
D	OXA-10	9 (±2) × 10^3^	3.4 (±0.1) × 10^–6^	70
D	OXA-23	5.1 (±0.2) × 10^3^	1.10 (±0.04) × 10^–5^	100
D	OXA-24	9 (±2) × 10^3^	1.7 (±0.1) × 10^–5^	80
D	OXA-48	8 (±2) × 10^5^	2.5 (±0.3) × 10^–5^	5 × 10^3^
D	OXA-58	2.5 (±0.3) × 10^5^	1.6 (±0.3) × 10^–4^	120 ± 40
D	OXA-66	6 (±0.7) × 10^2^	NT	NT

*^*a*^Values shown are averages ± standard deviations, or single measurements. The *k_*inact*_/K_*i*_* parameter is described in Tonge (2019). Measurements are from Durand-Reville et al. (2017), Shapiro and Gao (2021); Shapiro et al. (2021)Barnes et al. (2019) and Vázquez-Ucha et al. (2017). NT, not tested.*

The potency of serine β-lactamase inhibition by durlobactam has been measured for several enzymes of Ambler classes A, C, and D (Durand-Reville et al., 2017; Barnes et al., 2019; Shapiro and Gao, 2021). The second-order rate constants *k_*inact*_/K_*i*_* (Tonge, 2019) for covalent inhibition of representative serine β-lactamases are shown in Table 1. Values for avibactam, the first approved DBO β-lactamase inhibitor, are shown for comparison. Durlobactam has greater potency than avibactam for class A and C enzymes. Unlike avibactam, durlobactam has potent activity against all class D β-lactamases studied, many of which are commonly involved in β-lactam resistance in *A. baumannii* (Poirel et al., 2010; Durand-Reville et al., 2017; Table 1).

The *k_*inact*_/K_*i*_* values for avibactam shown in Table 1 were measured under identical conditions as those for durlobactam. Ehmann et al. (2013) also reported *k_*inact*_/K_*i*_* for avibactam with CTX-M-15, TEM-1, KPC-2, *Pseudomonas aeruginosa* AmpC, *Enterobacter cloacae* P99, OXA-10 and OXA-48. Those measurements differ by less than one order of magnitude from the values in Table 1.

Upon dilution of the β-lactamase-durlobactam complex into the pM concentration range, most enzymes recover some or all of their catalytic activity, showing that the inhibitor can dissociate from the enzyme. The rate constant for dissociation (*k*_*off*_) of durlobactam varies between β-lactamases (Shapiro et al., 2017) as shown in Table 1, with the lowest values observed with class D enzymes.

**SCHEME 1:**
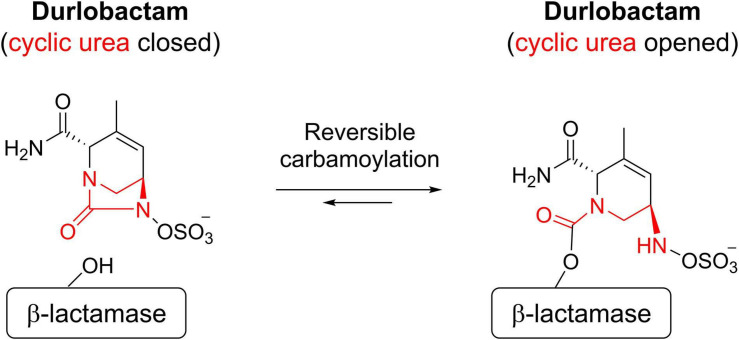
Mechanism of action of durlobactam.

When durlobactam reacts with a β-lactamase, the enzyme is carbamoylated on the active site serine nucleophile with the full mass of the inhibitor (277 Da) and the cyclic urea is opened (Scheme 1).

The covalent bond formed between durlobactam and the active site serine, like that of avibactam (Ehmann et al., 2012), is reversible because the sulfated amine is able to recyclize onto the carbamate, allowing the intact inhibitor to dissociate (Scheme 1).

The demonstration that intact durlobactam dissociates, rather than being released as a hydrolytic product, is the ability of the inhibitor to exchange from one enzyme molecule to another (acylation exchange) (Shapiro et al., 2017). Since durlobactam with the cyclic urea opened is not reactive with β-lactamases, acylation exchange shows that the ring reforms. However, partial loss of 80 Da from the mass of the acyl-enzyme complex, probably due to loss of the SO_3_ moiety, was observed with a subset of β-lactamases (KPC-2, *E. cloacae* P99 and OXA-10). This modification likely leads to an irreversibly inhibited enzyme, since the 197 Da adduct remained in place during acylation exchange experiments.

The average number of molecules of durlobactam per molecule of β-lactamase required to achieve full inhibition, which is known as the partition ratio or turnover number, was measured for several enzymes (Shapiro et al., 2017). In most cases, the turnover number was approximately 1, demonstrating that there was no detectable hydrolysis of durlobactam by the enzymes. An exception was KPC-2. The KPC-2 turnover number slowly increased with time, from 1.5 after a 15-min incubation to 3.0 after a 2-h incubation (Shapiro et al., 2017). This shows that KPC-2 is capable of very slowly hydrolyzing durlobactam, requiring about an hour for a single turnover. An even slower rate of hydrolysis was observed with OXA-10. Such slow rates of durlobactam hydrolysis are unlikely to significantly affect the utility of the inhibitor against these enzymes in whole cells.

In addition to inhibiting β-lactamases, some DBO β-lactamase inhibitors also exhibit intrinsic antibacterial activity due to inhibition of PBP2 (Morinaka et al., 2015; Moya et al., 2017; Miller et al., 2020). Durlobactam predominantly inhibits PBP2 of *A. baumannii* with a *k_*inact*_/K_*i*_* value of 1,800 ± 600 M^–1^ s^–1^ (Durand-Reville et al., 2017). Further evidence for the inhibition of PBP2 by durlobactam is shown in micrographs of *A. baumannii* treated with sub-minimal inhibitory concentration (MIC) concentrations of durlobactam (Durand-Reville et al., 2017). The round shape upon treatment of the normally rod-shaped bacteria is indicative of PBP2 inhibition (Nonejuie et al., 2013). Whereas PBP2 inhibition by durlobactam results in intrinsic antibacterial activity *in vitro* against *Escherichia coli* and several other *Enterobacterales* species, it has little to no effect on the growth of *A. baumannii* or *P. aeruginosa* when administered alone (Durand-Reville et al., 2017).

A polar compound such as durlobactam most likely enters Gram-negative cells through outer membrane porins. Iyer et al. (2018) showed that durlobactam enters *A. baumannii* cells through OmpA, the major and most abundant porin. Virulence of an *A. baumannii* strain in a mouse infection model was greatly impaired when OmpA was deleted, suggesting that loss of the porin is an unlikely mechanism for developing resistance to durlobactam (Iyer et al., 2018).

Due to its potent, broad-spectrum inhibition of serine β-lactamases, durlobactam restores the susceptibility of contemporary *A. baumannii* clinical global isolates to sulbactam (Durand-Reville et al., 2017; Barnes et al., 2019). Durlobactam also restores sulbactam susceptibility to engineered *A. baumannii* strains overexpressing individual β-lactamases (Durand-Reville et al., 2017).

## Structural Analysis of Durlobactam

The X-ray crystal structure of durlobactam in covalent complex with OXA-24/40 at 2.0 Å resolution (PDB: 6MPQ) was solved by Barnes et al. (2019; Figure 1B). The structure shows the covalent bond with the active site serine S81 side chain and the open urea ring. The carbonyl oxygen occupies the oxyanion hole formed by the backbone nitrogen atoms of S81 and W221. The sulfate group interacts with R261, S219, and S128 and two water molecules. S128 also interacts with the nitrogen atom that is bound to the sulfate group. The methyl group of durlobactam was introduced to interact with the “hydrophobic bridge” formed by Y112 and M223 (Durand-Reville et al., 2017) and the structure confirms that this design was successful. The methyl group forms a hydrophobic interaction with the side chain of M223. The amide group forms hydrogen bonds with a backbone carbonyl of W221 and three water molecules. The side chains of Y112, W115, W221, M223, and V130 form a hydrophobic pocket for the tetrahydropyridine ring.

## Sulbactam-Durlobactam Has Potent Activity Against *Acinetobacter* Spp. *in vitro* and *in vivo*

Contemporary MDR *A. baumannii* isolates are remarkable in the number and diversity of β-lactamase genes each individual strain encodes. A recent analysis of 84 non-clonal, globally diverse clinical isolates from 2006 to 2014 revealed that all strains encoded at least two and up to five distinct β-lactamase genes. These included endogenous class C *adc* (*Acinetobacter*-derived cephalosporinase) β-lactamases plus at least one and up to three distinct class D β-lactamases. In addition, over half of the isolates also encoded at least one and sometimes multiple class A β-lactamase genes. This analysis confirmed that any effective *Acinetobacter*-targeting BLI must demonstrate potent, broad activity against all three classes of serine β-lactamases. The meropenem MIC_90_ against this collection of strains was ≥128 mg/L whereas the sulbactam-durlobactam MIC_90_ was 4 mg/L (Durand-Reville et al., 2017).

Similarly potent *in vitro* activity of sulbactam-durlobactam has been demonstrated by a number of surveillance studies on recent MDR *A. baumannii* isolates from around the world (McLeod et al., 2020; Seifert et al., 2020) including China (Yang et al., 2020) and South America (Nodari et al., 2021), with MIC_90_ values ranging from 2/4 to 4/4 mg/L. The potency was maintained against all subsets of resistant phenotypes, including carbapenem-resistant, colistin-resistant and MDR/extremely drug-resistant (XDR) subsets (McLeod et al., 2020). The rare isolates with elevated sulbactam-durlobactam MICs were found to encode either PBP3 mutations near the sulbactam binding site or metallo-β-lactamae *bla*_*NDM–*__1_, which durlobactam does not inhibit (McLeod et al., 2020). Notably, the most common PBP3 mutations found in clinical isolates with elevated sulbactam-durlobactam MICs were A515V and T526S (Figure 1A; McLeod et al., 2020). No clinical isolate has yet been found to encode either of the aforementioned laboratory-generated, sulbactam-resistant S390T or S395F PBP3 variants (Penwell et al., 2015), supporting the hypothesis that these mutations may confer a fitness cost to the organism and therefore are unlikely to spontaneously occur during treatment with sulbactam-durlobactam

The *in vivo* efficacy of sulbactam-durlobactam has been demonstrated at clinically relevant exposures in numerous thigh and lung murine infection models against XDR *A. baumannii* clinical isolates with sulbactam-durlobactam MIC values ranging from 0.5/4 to 4/4 mg/L (Durand-Reville et al., 2017; Barnes et al., 2019). The combination also showed excellent preclinical safety (Durand-Reville et al., 2017) before advancing to clinical testing several years ago.

## Discussion

Significant efforts devoted to the discovery of novel BLIs over past decades have led to important breakthroughs in the field. In particular, the new mechanisms of inhibition and spectra of activity of the non-β-lactam DBO and boronate classes (Bush and Bradford, 2019) have led to United States Food and Drug Administration (FDA) approvals for several new β-lactam/β-lactamase inhibitor therapies for MDR Gram-negative bacteria (Torres et al., 2018; Zhanel et al., 2018; McCarthy, 2020). However, none of these are active against carbapenem-resistant *Acinetobacter* infections. In contrast, extensive preclinical evidence suggests sulbactam-durlobactam is highly effective against these problematic pathogens.

Sulbactam-durlobactam was well tolerated in Phase 1 studies in healthy volunteers, and in a Phase 2 study in combination with imipenem in patients with complicated urinary tract infections, including acute pyelonephritis (O’Donnell et al., 2019; Sagan et al., 2020). Sulbactam-durlobactam is currently being evaluated in a pivotal, pathogen-targeted, global Phase 3 trial (called ATTACk, for **A**cinetobacter **T**reatment **T**rial **A**gainst **C**olistin), to determine its efficacy and safety in patients with bloodstream infections, hospital-acquired or ventilator-associated bacterial pneumonia due to *A. baumannii-calcoaceticus* complex (registered at ClinicalTrials.gov under the identifier NCT03894046). If the phase 3 trial confirms clinical efficacy and safety and gains regulatory approval, sulbactam–durlobactam will be an important treatment option for patients with serious and life-threatening infections caused by *Acinetobacter* species, including carbapenem-resistant strains.

## Author Contributions

AS, SMM, and AM wrote sections of the manuscript. SHM generated the computational model shown in Figure 1A and provided comments on the manuscript. TD-R created Scheme 1 and provided comments on the manuscript. All authors contributed to the article and approved the submitted version.

## Conflict of Interest

All co-authors are employees of Entasis Therapeutics. The authors declare that this study received funding from Entasis Therapeutics. The funder had the following involvement in the study: the study design, collection, analysis, interpretation of data, the writing of this article and the decision to submit it for publication.
